# Exploring How the Home Environment Influences Eating and Physical Activity Habits of Low-Income, Latino Children of Predominantly Immigrant Families: A Qualitative Study

**DOI:** 10.3390/ijerph15050978

**Published:** 2018-05-14

**Authors:** Ana Cristina Lindsay, Sherrie F. Wallington, Faith D. Lees, Mary L. Greaney

**Affiliations:** 1 Department of Exercise and Health Sciences, University of Massachusetts–Boston, 100 Morrissey Boulevard, Boston, MA 02125, USA; 2 Department of Nutrition, Harvard T.H. Chan School of Public Health, Boston, MA 02115, USA; 3 Lombardi Comprehensive Cancer Center, Georgetown University Medical Center, Washington, DC 20057, USA; slw49@georgetown.edu; 4 Program in Gerontology, University of Rhode Island, Kingston, RI 02881, USA; flees@uri.edu; 5 Health Studies & Department of Kinesiology, University of Rhode Island, Kingston, RI 02881, USA; mgreaney@uri.edu

**Keywords:** home environment, Latino, obesity, parents, healthy eating, physical activity

## Abstract

Latinos are the largest and fastest growing minority population group in the United States, and children in low-income Latino families are at elevated risk of becoming overweight or having obesity. A child’s home is an important social environment in which he/she develops and maintains dietary and physical activity (PA) habits that ultimately impact weight status. Previous research suggests the parents are central to creating a home environment that facilitates or hinders the development of children’s early healthy eating and PA habits. Therefore, the purpose of this study was to explore low-income Latino parents’ beliefs, parenting styles, and parenting practices related to their children’s eating and PA behaviors while at home. Methods: Qualitative study using focus group discussions (FGDs) with 33 low-income Latino parents of preschool children 2 to 5 years of age. FGDs were transcribed verbatim and analyzed using thematic analysis. Results: Data analyses revealed that most parents recognize the importance of healthy eating and PA for their children and themselves. However, daily life demands including conflicting schedules, long working hours, financial constraints, and neighborhood safety concerns, etc., impact parents’ ability to create a home environment supportive of these behaviors. Conclusions: This study provides information about how the home environment may influence low-income Latino preschool children’s eating and PA habits, which may be useful for health promotion and disease prevention efforts targeting low-income Latino families with young children, and for developing home-based and parenting interventions to prevent and control childhood obesity among this population group. Pediatric healthcare providers can play an important role in facilitating communication, providing education, and offering guidance to low-income Latino parents that support their children’s development of early healthy eating and PA habits, while taking into account daily life barriers faced by families. Moreover, pediatric healthcare providers also can play an important role in the integration and coordination of home-visitations to complement office-based visits and provide a continuum of care to low-income Latino families.

## 1. Introduction

Latinos are the largest and fastest growing minority population group in the United States (US) [1], and children in low-income Latino families are at elevated risk of becoming overweight and having obesity [2]. Research shows that racial/ethnic disparities in weight status among Latino children of immigrant families living the US are associated with socioeconomic disparities (e.g., lower maternal education and family income) [3,4]. Furthermore, Latino children from primarily Spanish-speaking households have higher mean body mass index (BMI) than white children and Latino children from English-speaking households [4]. Additional factors contributing to Latino children’s elevated risk of obesity include food insecurity, poverty, and parental unemployment [5]. Eight percent of Latinos live in deep poverty (income below 50% of the federal poverty threshold) compared to 6% of all people in the US [6], and one in five (20%) Latinos are food insecure compared to one in 10 (9.5%) non-Hispanic whites [7]. Food insecurity describes a household’s inability to provide enough food for every person to live an active and healthy life [8]. Poverty and food insecurity are important social and system determinants that can lead to disparities in health [5].

Eliminating disparities in weight status among Latino children of immigrant families requires a deeper understanding of the early life influences prevalent in low-income Latino homes to identify factors relevant to this population group that are amenable to intervention [3,4]. Christensen’s [9] ‘health-promoting family’ framework depicts not only genetic background, but also aspects of the ‘family eco-cultural pathway’ (values, goals, needs, health practices) as proximal influences on children’s health behaviors that mediate community and societal influences. This framework emphasizes the expression of cultural pathways in terms of everyday practices and routines [9]. Moreover, the family has been described as “a setting of health practice” at the center of a network of social systems [10], highlighting parents’ role in integrating interactions with people, organizations, and the community that may affect their children’s lifestyle behaviors [10]. Family plays a central role in Latino culture, and traditional Latino family values, often referred to as familism, emphasize the centrality of the family unit and stress the obligations and support that family members owe to both their nuclear family and extended kin [11]. According to familism values, children’s behaviors are a reflection of the family unit and as a result parents often direct their children to behave in a way that positively reflects their family values [11]. Latino familism provides a lens for examining the cultural impact on weight status [12].

A child’s home is an important social environment in which he/she develops and maintains eating and physical activity (PA) habits that ultimately impact weight status [4,13,14,15]. Previous research suggests that parents are central to creating a home environment that facilitates or hinders the development of children’s healthy eating and PA habits [4,13,16,17,18]. Parents provide not only the genetic predisposition to obesity, but also serve as role models and create home environments that shape their children’s experiences with eating and PA [17,19]. The home environment includes among other characteristics, parents’ own eating and PA behaviors (i.e., modeling of behaviors) as well as parenting styles and parenting practices related to children’s eating, PA and sedentary habits [4,20,21].

Parental modeling of healthy eating and PA and sedentary habits may be particularly influential in young children developing lifelong healthy eating and PA behaviors that contribute to healthy weight status [22,23,24]. Prior research suggests that parenting styles influence childhood obesity [18,21,25,26]. Parenting styles encompass the overarching attitudes and behaviors that define how parents interacts with their children across several aspects of parenting, including the physical, social, intellectual, emotional, etc. components parenting [27,28]. Two primary dimensions of parenting styles have been identified: (1) demandingness, which is defined as control, and (2) responsiveness, also defined as warmth. Combined, demandingness and responsiveness are used to describe four types of parenting styles: (1) authoritarian (high demandingness/low responsiveness); (2) authoritative (high demandingness/high responsiveness); (3) permissive (low demandingness/high responsiveness); and (4) uninvolved (low demandingness/low responsiveness) [25]. Research indicates that children raised with authoritative parenting style consume a more healthful diet, participate in more PA and have a lower BMI levels than children raised with other parenting styles (authoritarian, permissive, uninvolved) [21]. Parenting practices are the specific sets of behaviors that characterize how parents interact on a daily basis with their children, and the beliefs and attitudes that underpin these interactions [25,27,28,29]. Parenting feeding (e.g., pressuring child to eat) and parenting PA (e.g., setting screen time limits) practices have been shown to influence children’s eating habits, PA behaviors, and weight status [19,27,28,29,30,31].

Currently, there is still a dearth of research examining low-income, Latino immigrant parents’ parenting styles and parenting practices related to children’s eating, PA and sedentary behaviors (SB). Therefore, the purpose of this qualitative study was to explore low-income Latino immigrant parents’ beliefs, parenting styles and parenting practices related to eating, PA and SB of their preschool-age children while at home in order to identify salient intervention targets and strategies for this population group.

## 2. Materials and Methods

### 2.1. Setting and Sample

The current study is part of a larger multicomponent qualitative research project exploring factors influencing eating, PA, and SB among low-income Latino children of preschool age (2–5 years) attending licensed family child care homes (FCCHs) in Massachusetts (MA) [20,32,33,34]. FCCHs are a type of early care and education (ECE) setting where providers care for children other than their own in their homes [35,36]. More than 1.9 million preschool children attend FCCHs making this ECE setting the second largest provider of non-relative care for children under the age of 5 years in the US [36].

This paper presents some of the results of exploratory focus group discussions (FGDs) that were conducted to gain an in-depth understanding of Latino parents’ perceptions of their children’s eating and PA behaviors while at home. FGDs were used because they are an important technique for conducting research in diverse cultural settings that provides rich and valuable information. Moreover, the synergistic effects of the group settings elicit ideas and discussion that may not arise in individual interviews. A convenience sample of low-income Latino parents was recruited through licensed Latino FCCHs participating in the larger study [34]. Participating FCCH providers enrolled in the larger multicomponent qualitative study assisted study staff in recruiting Latino parents with children between 2–5 years of age attending their FCCH to participate in this current study. Parents were invited to participate in the current study if they self-identified as Latino, had at least one child between 2 and 5 years old attending one of the selected licensed FCCHs, and had lived in the US for at least 12 months.

### 2.2. Data Collection

Six focus FGDs with Latino parents (27 mothers and six fathers, representing 33 unique families) of preschool children were conducted in meeting rooms of public libraries between April and September 2015. A native Spanish speaker trained in qualitative research methods moderated all FGDs in Spanish using the same discussion guide for all FGDs. The guide had open-ended questions and probes has been described in detail elsewhere [20]. In brief, the pilot-tested FGD guide explored (a) parents’ beliefs and attitudes related to eating, PA and SB; (b) parents’ perceptions of children’s eating and PA experiences at home; (c) parenting practices related to eating, PA and SB behaviors at home; and (d) barriers faced in structuring a home environment conducive to healthy eating and PA. The FGD guide was pre-tested with a small group of Latino parents (*n* = 4) to assess the understanding and flow of the questions. Findings of the pilot FGD were used to refine the final guide including changing the structure of some questions, adding additional probes, and changing the questions order in some parts of the guide.

Each digitally recorded FGD lasted between 60 and 80 min. At the start of the FGDs, the moderator directed participants to think about their oldest preschool-age child (2–5 years of age) when participating in the discussion. After the FGDs, participants completed a brief, self-administered questionnaire that assessed education, marital status, country of origin, length of time living in the US, and acculturation, which was assessed via the Short Acculturation Scale for Hispanics [37], a 12-item measuring scale validated for use in Latino groups such as Mexican Americans, Cuban Americans, Puerto Ricans, Dominicans, and Central and South Americans. The study was approved by the Institutional Review Board for the Protection of Human Subjects at the Harvard T. H. Chan School of Public Health (#259231), and all participants provided signed informed consent and received a $25 gift card for their participation.

### 2.3. Data Analysis

The first step of the analysis was to transcribe the Spanish recordings verbatim in Spanish. The transcripts were then translated into English with all identifiers removed. A professional bilingual transcriptionist then translated the transcripts using forward-backward techniques to establish semantic equivalence in translation [38]. This technique was used to ensure that the integrity of the qualitative data was not lost during translation [38]. Translated transcripts were analyzed using thematic analyses, an iterative process of coding data in phases to identify meaningful patterns [38,39]. Two authors, (FL, ACL) employed a multiphase analytic process that included becoming familiar with the data, generating initial codes, coding all transcripts, checking for coding consistency, resolving coding discrepancies, identifying themes and patterns, and defining and naming themes [39]. Microsoft Excel 2008^®^ was used to calculate the descriptive statistics from the socio-demographic data collected.

## 3. Results

A total of six FGDs were conducted before thematic saturation occurred, with no new information emerging during the sixth focus group. Each FGD had an average of six participants (range: 4–7). Participants’ (*n* = 33) mean age was 29 years (range: 26 to 37 years; see Table 1). Participants were originally from South America (e.g., Colombia, Peru), Central America (e.g., Guatemala, Honduras, El Salvador), Mexico, or the Caribbean (e.g., Dominican Republic, Puerto Rico). Approximately 73% (*n* = 24) of participants were married. Participants had approximately two children on average, and about 77% (*n* = 26) reported being undocumented U.S. residents. More than half of participants (61%, *n* = 20) had not graduated from high school, and 18.2% (*n* = 6) were unemployed. Approximately two-thirds (70%; *n* = 23) reported a family income ≥$20,000 and <$40,000. Participants had lived in the US for an average of 9 years (SD = 2.4), and participants more closely identified with Latino culture, although their mean acculturation score of 2.3 (SD = 0.7) was close to “bicultural”. 

The analysis identified four main themes and these themes are presented below with illustrative quotes.

**Theme** **1.** *Parents know that healthy eating and physical activity are important*.

Across all FGDs, parents reported strong beliefs about the importance of children eating healthy and being physically activity. One parent mentioned,

We all know that eating healthy and being physically active is very important for children to grow healthy and develop good habits early on. I believe my responsibility is a mother is to teach my children to be healthy. (Colombian mother, two children—a 4-year-old daughter and a 10-year-old son)

Additionally, parents spoke of the importance of children developing these habits when young. As one parent explained,

I try to teach my children the importance of eating healthy and being active. I believe children should learn and develop these habits early in life to help them grow healthy and get used to healthy habits. (Colombian father, two children—a 5-year-old son and an 11-year-old daughter)

Nonetheless, several parents reported that despite their continued efforts and best intentions to instill healthy eating and PA habits in their young children, their children might not eat as healthfully and be as physically active at home as they would like. One parent noted,

I try to pay attention to the food I buy and try to cook healthy foods at home. I know it’s important that they [children] eat well and are physically active, but it’s not always easy to do it. Sometimes the kids are picky and don’t want to eat what I cook ... finding time to take the kids outside to play after a long day of work is also not always possible. (Colombian mother, two children—a 5-year-old son and a 9-year-old daughter)

Another parent reported,

With my daughter is a constant “battle”. I like for her [daughter] to eat more vegetables. She doesn’t like vegetables. She says I have to add some dressing so she can eat the vegetables ... she doesn’t like the taste. (Colombian mother, one child—a 4-year-old daughter)

**Theme** **2.** *Parents want to be role models but struggle with eating healthfully and being physically active themselves*.

Parents felt that they play an influential role in helping their children eat healthfully, be physically active, and, eventually, become healthy adults. One parent mentioned,

We all need to role model good eating habits for our children ... we should show portion control as adults and also not eat what children leave on their plates. (Dominican mother, one child—a 4-year-old daughter)

Another parent added,

I try to show my son that it’s important to be physically active. We play soccer together and I encourage him to play in our local soccer league. (Colombian father, two children—a 5-year-old son and an 11-year-old daughter)

Although the majority of parents viewed their own eating and PA behaviors as influencing those of their children, they spoke of the challenges of modeling these behaviors and the need to improve their own eating and PA habits. One parent noted,

I know it’s important that we [parents] also eat healthy and are active. We need to set a good example for our children. I am always trying to eat healthy—fruits and vegetables ... but, we [parents] also make mistakes. It’s important to keep trying. (Puerto Rican mother, two children—a 3-year-old son and a 6-year-old daughter)

Another parent added,

It is not always easy to do what you know is right. You are tired and still have a lot to do, so it’s easy to go for fast food and just think that it’s only one day ... but I think it is important to keep trying. (Ecuadorian mother, two children—a 5-year-old daughter and a 3-year-old son)

**Theme** **3.** *Parents face socioeconomic and logistical barriers in helping their children eat healthfully and be physically active while at home*.

Most parents stated that they would like their children to eat healthier at home (e.g., eat more fruits and vegetables and fewer sweets). Nearly all parents, however, spoke of day-to-day barriers such as time pressures, conflicting working schedules, financial constraints, that make it difficult for their children to eat healthfully and/or be physically active while at home. One parent stated,

During the week, I am busy with work and have less time, so usually the kids eat first. I try to prepare something quick, give them a bath and put them in bed. During the weekends, I try to make some time for cooking, and on Sundays especially we eat together as a family. That’s when we have more time. (Colombian mother, two children—a 4-year-old daughter and a 10-year-old son)

Moreover, many parents reported that their children engage in limited PA and excessive screen time (e.g., television viewing and other electronic devices) while at home due to parents’ competing demands, neighborhood safety concerns, limited space within their home, and cold weather. One parent reported,

My son is always in his iPad playing video games and watching movies. It’s a battle to get him to do anything else, especially when we get home and my wife still needs to make dinner and get everything ready for the next day. (Colombian father, two children—4-year-old some and 9-year-old son)

One parents added,

By the time I get home and have to get dinner ready there isn’t much time left to take the kids outside to play. They end up sitting and watching some TV while I get things done. (Dominican mother, two children—a 4-year-old daughter and a 7-year-old son)

Another parent mentioned,

I don’t feel safe letting the kids go outside and play by themselves. An adult needs to be with them at all times. The streets are not safe. My husband doesn’t get home until later and most days the kids are already in bed by the time he gets home. (Puerto Rican mother, three children—4-year-old son, 7-year-old-son, and a 9-year-old daughter)

Furthermore, several parents spoke of difficulty overcoming daily life demands—long working hours, conflicting working schedules, and limited household budgets—to make changes that they believed would result in their children having healthier eating and PA behaviors while at home. One parent mentioned,

It would be good if we could enroll them in some kind of program, especially during the winter, when they don’t spend a lot of time outside, but by the time we pay the bills and buy food, the money is all gone. (Dominican mother, two children—a 4-year old daughter and a 7-year-old son)

**Theme** **4.** *Many parents report permissive parenting styles*.

The majority of parents reported struggling with enforcing healthy eating at home. For instance, several parents reported that they often consented to their children’s demands for foods that were not the best choice, indicative of permissive feeding style. One parent reported,

I get home and I am tired. We have been apart for the whole day. I don’t like to get into fights about what my daughter wants to eat or not. So, most of the time she eats what she likes … and sometimes it’s not as healthy as I would like it to be. Like, I’d like her to eat more vegetables. (Dominican mother, one child—a 5-year-old daughter)

Another parent stated,

My problem with my son is that he is always eating snacks. I try not to buy a lot of junk food, but the kids beg me and I ended up buying some, and if you have those foods at home, there is no way to keep the kids from eating them. (Peruvian mother, two children—a 6-year-old son and a 4-year-old daughter)

Moreover, the majority of parents described not setting limits or having rules around screen time for their children, also indicative of a permissive parenting style. One parent mentioned,

At our house, we don’t really have rules about how much TV or video games the kids can or cannot play. I get home with the kids at around 4:00 PM and my husband gets home at around 4:30 PM. He [husband] gets home and the first thing he does is to turn the TV on ... the kids watch TV with him and play inside the house while I get things ready for dinner. (Salvadoran mother, two children—a 4-year-old son and a 7-year-old daughter)

Another parent reported,

At home the children watch a lot of TV. I sometimes try to set limits, but I need to get the work done around the house, prepare dinner, and it helps me to have them occupied. (Guatemalan mother, three children—4-year-old son, 10-year-old son, and an 8-year-old daughter)

Another parent mentioned,

My daughter gets home and the first thing she asks for is to watch TV ... she’s not allowed to watch TV at the daycare, so I let her watch TV while I cook, sometimes even when she’s eating dinner and while I clean things up. (Ecuadorian mother, one child—a 5-year-old daughter)

## 4. Discussion

Racial and ethnic disparities in overweight and obesity are associated with differences in socioeconomic factors and health disparities (e.g., lower maternal education, family income, food insecurity, etc.) that shape children’s home environment and household routines [4,40,41,42,43,44]. Parents are central to developing a home environment conducive of children’s development of healthy eating and PA behaviors [17]. Prior research suggests that several characteristics of the home environment including parental modeling, parenting styles and parenting practices influence children’s eating, PA, SB, and weight status [4,16,22,24,30,44,45,46,47,48,49,50,51,52,53,54,55,56]. Therefore, the present qualitative study was designed to explore low-income Latino parents’ beliefs, parenting styles and parenting practices related to healthy eating and PA of their children while at home.

Most parents participating in the current study recognized the importance of healthy eating and PA for their children, but reported that conflicting schedules, long working hours, financial constraints, and neighborhood safety concerns impacted their ability to create a home environment supportive of these behaviors. Although it is essential that parents recognize the importance of healthy behaviors, parents must have the means and capacity to create changes to their home and family environments to support their children’s healthy eating and PA behaviors [41,42,44,46]. Changing the home food and PA environments of low-income families from minority backgrounds may involve additional considerations of socioeconomic barriers to facilitate improved behaviors in this setting [44,45,46,47]. Successful home-based interventions should take into account parents’ beliefs and intentions, and work with parents to positively reconcile differences in these beliefs and intentions with the day-to-day difficulties and pressures faced by low-income families. 

Study findings revealed that low-income Latino parents participating in the present study had difficulties setting family routines conducive and supportive of their young children’s healthy eating and PA behaviors. This is an important finding with intervention implications as research shows that children living in households with regular family routines (e.g., regularly eating meals as a family) are at lower risk of obesity compared to children living without household routines [41].

Similar to prior research with Latino parents [57,58], we found that low-income Latino parents in our study struggled with modeling healthy eating and PA habits for their young children. This is noteworthy as social cognitive theory posits that behaviors are influenced by many factors, one of which is observational learning [59,60]. Therefore, improving Latino parents’ eating and PA behaviors would not only be beneficial for the parents’ own health status, but would also be an important target in the promotion of Latino children’s healthy eating and PA behaviors.

Consistent with prior studies [51,52,53], we found that parents reported permissive parenting styles associated with children’s unhealthy eating such as allowing unhealthy food choices, and sedentary activities such as excessive screen time behaviors. A longitudinal study of Mexican-American mothers and their children showed that children’s whose parents displayed a more permissive parenting style related to children’s diet were at increased risk of being overweight during the next three years compared with those having authoritative or authoritarian parents [52]. A more recent qualitative study conducted with Mexican-American and Mexican-immigrant mothers and fathers revealed that both fathers and mothers were very permissive of their children’s unhealthy food choices, and that mothers were more permissive than fathers about children’s engagement in sedentary activities [53]. Taken together these results suggest the need for interventions targeting parenting styles and the home environment of minority, low-income Latino children. Yet, to date, there is a dearth of interventions targeting parenting styles of low-income, Latino parents of preschool-age children [54,55,56].

Furthermore, Latino parents participating in our study struggled with implementing parenting practices supportive of their children’s healthy eating and PA (e.g., consumption of unhealthy foods and not setting limits on screen time). Given that a growing body of research suggests that parenting practices influence children’s healthy eating and PA behaviors it is important that parents be provided the resources needed to develop these parenting skills [48,49,50].

Previous research has documented the home environment to be an important target of multi-level interventions [22,44]. Findings of our study suggest that interventions addressing overweight and obesity prevention in young low-income Latino children should focus on parental behaviors, parenting styles and parenting practices within the immediate home environment. Our study revealed that low-income, Latino parents face economic and logistical barriers associated with the day-to-day pressures and challenges of low socioeconomic minority families. These findings suggest that parenting interventions that build parent capacity and self-efficacy framed within a broader socio-ecological model that address multiple barriers that hinder Latino parents’ ability to support their children’s healthy eating and PA are needed [10,16,22]. Prior research suggests that parent support programs that are family centered and provide capacity building are effective ways to deliver support and information to help parents become more capable and competent in creating a healthful home environment [61,62,63].

Given pediatric healthcare providers frequent and continued contact with parents and children throughout the early childhood years through well-child visits, they are well positioned to work with parents to promote and support the development of healthy early eating and PA behaviors of young Latino children. Pediatric healthcare providers can play an important role in facilitating communication and offering guidance to parents about specific ways to help their children develop early healthy eating and PA habits, while taking into account daily life barriers faced by low-income Latino families. Pediatric healthcare providers can work closely with parents to help them identify, develop, and prioritize family and household routines (e.g., family dinners) conducive of these early healthful behaviors. Furthermore, pediatric healthcare providers also can play a critical role in helping parents develop parenting skills (e.g., limit setting) necessary to the implementation of successful family routines. Finally, pediatric healthcare providers can play an important role in the integration and coordination of home visitations to complement office-based visits and provide a continuum of care to low-income Latino families. Home visitations may offer an effective mechanism to ensure ongoing parental education, social support, and linkage with public and private community services that can facilitate and support the development of early healthy eating and PA habits of low-income Latino children, and ultimately the prevention of obesity and related chronic diseases [63].

Findings of the present study should be considered in light of some limitations, including a small sample size, and the use of purposive sampling needed to enroll low-income, Latino parents in MA into the study, which limit generalizability. The present study was designed to explore parents’ perceptions of and practices related to their children’s eating and PA environments while at home, and not to determine children’s dietary intake or PA levels while at home. Furthermore, study participants may have chosen to participate in the study due to their interest in PA and/or diet, which may further limit generalizability. Nevertheless, the study provides insight into personal perceptions and practices of low-income, predominantly immigrant Latino parents concerning their children’s eating and PA experiences while at home.

## 5. Conclusions

This qualitative study provides information on how the home environment influences low-income, Latino preschool children’s eating and PA habits that can be used to assist in health promotion and disease prevention efforts designed for low-income Latino families of young children, and in the development of interventions to prevent and control childhood obesity among this population.

Further research is needed to examine the complex inter-relationships of the home environment within the broader contextual influences (e.g., economic environments) that ultimately impact child health outcomes such as healthy eating and PA behaviors. For example, future studies should explore the impact of work, socioeconomic status, food insecurity, parent role modeling, and parental capacity of low-income Latino parents to make changes conducive to healthy eating and PA behaviors for their children and family. Understanding social contextual factors that influence low-income, Latino parents’ beliefs, behaviors, parenting styles and parenting practices related to healthy eating and PA habits will enhance efforts to address Latino children’s unhealthy eating and PA habits.

## Figures and Tables

**Table 1:** Socio-demographic and acculturation characteristics of focus group participants (*n* = 33).

	Mean ± SD	*n* (%)
Age *	29 ± 2.3	33 (100)
Race		
Hispanic or Latino		33 (100)
Foreign-born		
Yes		31 (93.9)
No		2 (6.1)
Country of origin		
Colombia		9 (27.3)
Dominican Republic		8 (24)
Guatemala		4 (12.1)
Puerto Rico (US territory)		3 (9.1)
Peru		3 (9.1)
United States		2 (6.1)
Mexico		2 (6.1)
El Salvador		1 (3.1)
Honduras		1 (3.1)
	**Mean ± SD**	
Years in the United States	9 ± 2.4	
Predominant language spoken at home		
Spanish		33 (100)
	**Mean ± SD**	
Marin scale acculturation score	2.3 ± 0.7	
Marital status		
Single		1 (3.1)
Married		24 (72.7)
Divorced/Separated		8 (24.2)
	**Mean ± SD (Range)**	
Number of children	2.2 ± 1.4 (1–4 children)	
Education level		
Less than high school		20 (60.6)
High school degree		8 (24.2)
GED **		4 (12.1)
Missing		1 (3.1)
Household annual income		
≥$20K/year <$40,000		23 (69.7)
<$20K/year		10 (30.3)

* Average age of parents was calculated for both mothers (*n* = 27) and fathers (*n* = 6) participating in focus group; ** General Education Development.
